# Extracellular Vesicles Derived from Three-Dimensional-Cultured Human Umbilical Cord Blood Mesenchymal Stem Cells Prevent Inflammation and Dedifferentiation in Pancreatic Islets

**DOI:** 10.1155/2023/5475212

**Published:** 2023-02-20

**Authors:** Eunwon Lee, Seungyeon Ha, Gyuri Kim, Jae Hyeon Kim, Sang-Man Jin

**Affiliations:** ^1^Division of Endocrinology and Metabolism, Department of Medicine, Samsung Medical Center, Sungkyunkwan University School of Medicine, Seoul 06351, Republic of Korea; ^2^Samsung Biomedical Research Institute, Samsung Medical Center, Seoul 06351, Republic of Korea; ^3^Department of Health Sciences and Technology, Samsung Advanced Institute for Health Sciences & Technology, Seoul 06351, Republic of Korea

## Abstract

It is unclear whether extracellular vesicles (EVs) from mesenchymal stem cells (MSCs) have a direct protective effect on pancreatic islets. In addition, whether culturing MSCs in three dimensions (3D) instead of a monolayer (2D) can induce changes in the cargo of EVs that facilitate the polarization of macrophages into an M2 phenotype has not been investigated. We sought to determine whether EVs from MSCs cultured in 3D can prevent inflammation and dedifferentiation in pancreatic islets and, if so, whether the protective effect is superior to that of EVs from 2D MSCs. Human umbilical cord blood- (hUCB-) MSCs cultured in 3D were optimized according to cell density, exposure to hypoxia, and cytokine treatment based on the ability of the hUCB-MSC-derived EVs to induce the M2 polarization of macrophages. Islets isolated from human islet amyloid polypeptide (hIAPP) heterozygote transgenic mice were cultured in serum-deprived conditions with hUCB-MSC-derived EVs. EVs derived from 3D hUCB-MSCs had more abundant microRNAs involved in M2 polarization of macrophages and had an enhanced M2 polarization ability on macrophages, which was optimized when the 3D culture condition was 2.5 × 10^4^ cells per spheroid without preconditioning with hypoxia and cytokine exposure. When islets isolated from hIAPP heterozygote transgenic mice were cultured in serum-deprived conditions with hUCB-MSC-derived EVs, the EVs derived from 3D hUCB-MSCs suppressed the expression of proinflammatory cytokines and caspase-1 in pancreatic islets and increased the proportion of M2-polarized islet-resident macrophages. They improved glucose-stimulated insulin secretion, reduced the expression of Oct4 and NGN3, and induced the expression of Pdx1 and FoxO1. The greater suppression of IL-1*β*, NLRP3 inflammasome, caspase-1, and Oct4 and induction of Pdx1 and FoxO1 were found in islets cultured with the EVs derived from 3D hUCB-MSCs. In conclusion, EVs derived from 3D hUCB-MSCs optimized for M2 polarization attenuated nonspecific inflammation and preserved *β*-cell identity of pancreatic islets.

## 1. Introduction

Intravenous injection of mesenchymal stem cells (MSCs) improves the function and survival of pancreatic islets and preserves *β*-cell identity in animal models of type 2 diabetes [1–3] and pancreatic islet transplantation [4–6]. An important mechanism of these benefits is the ability of MSCs to induce polarization of macrophages into the M2 subtype. In type 2 diabetes and primary islet graft failure after pancreatic islet transplantation, the progression of inflammatory reactions such as the increase in islet macrophage infiltration, the polarization of macrophages into the M1 subtype, and the inflammasome activation play an essential role in the progression of *β*-cell failure [7]. Clinical trials on the infusion of MSCs into patients with type 2 diabetes [8] and recipients of pancreatic islet autotransplantation have been conducted without severe adverse events [9].

The use of MSC-derived extracellular vesicles (EVs) offers a promising alternative to MSCs themselves by reproducing their biological function in delivering nucleic acids, proteins, and lipids to the local microenvironment of damaged cells or tissues [10, 11]. Moreover, EVs could decrease safety concerns because they are nonimmunogenic and not likely to cause maldifferentiation [12, 13], which is a clinical concern of therapies using MSCs. Interestingly, it has been suggested that EVs derived from MSCs, without additional effects from MSCs themselves or other components of their secretome, can improve pancreatic *β*-cell survival and insulin sensitivity in rodents with low-dose streptozotocin and high-fat diet-induced diabetes [14]. This indicates that the use of MSC-derived EVs could be a novel approach to delaying the progression of *β*-cell failure in type 2 diabetes and primary islet graft failure after pancreatic islet transplantation.

MSCs cultured using the three-dimensional method (3D MSCs), which better reflects an *in vivo* environment, consistently exhibit enhanced anti-inflammatory, angiogenic, and tissue reparative/regenerative effects with improved cell survival after transplantation [15]. Such benefits are mediated, at least in part, by EVs from MSCs [16]. In addition to the changes in gene expression profiles such as the expression of genes with cell stemness and migration ability in 3D MSCs, EV production increases with the three-dimensional culture method, and the resulting EVs can have a cargo profile that is different from 2D MSCs [17]. In a recent study, total RNA sequencing using next-generation sequencing platforms on human amnion-derived 2D and 3D MSCs revealed profound transcriptome changes, including enhanced secretion of C-C motif chemokine ligand 2 (CCL2), C-X-C motif chemokine ligand 12 (CXCL12), and bone morphogenetic protein 2 (BMP2), which could contribute to a microenvironment favouring polarization of macrophages into the M2 phenotype [18]. However, a previous study conducted in an animal model of bleomycin-induced lung fibrosis suggested that EVs produced from 3D MSCs did not demonstrate enhanced immunomodulatory properties compared with 2D MSC-derived EVs [19], indicating that optimization of 3D MSC-derived EVs in each disease model is required.

EVs from cytokine-preconditioned MSCs contain several microRNAs (miRs) that can induce macrophage polarization into the M2 subtype, in contrast to EVs from resting MSCs [20, 21]. However, whether the cargo changes in EVs derived from 3D MSCs include an enhanced capacity for the polarization of macrophages into the M2 phenotype has not been investigated, and there has been no specific study that compared the benefits of EVs from either 2D or 3D MSCs on pancreatic islets. Although it has been suggested that intraperitoneal injection of 3D MSCs instead of 2D MSCs in a multiple low-dose streptozotocin-induced diabetes model better attenuates inflammatory processes in pancreatic islets and improves glycemic control [22], the role of 3D MSC-derived EVs in this benefit, if any, has not been determined.

Therefore, we investigated whether EVs from MSCs cultured in 3D have direct protective effects against inflammation and dedifferentiation on pancreatic islets and, if so, whether the protective effects are superior to those of EVs from monolayer-cultured MSCs (2D MSCs).

## 2. Materials and Methods

### 2.1. Animals and Cell Culture

Mouse islets were isolated from heterozygous human islet amyloid polypeptide (hIAPP) transgenic (hIAPP^+/-^) 8-12-week-old FVB/N mice (Jackson Laboratory, Bar Harbor, ME, USA). The Institutional Animal Care and Use Committee of Samsung Biomedical Research Institute approved all animal experimental protocols in this study. Human umbilical cord blood- (hUCB-) MSCs were isolated according to a reported method [23]. Umbilical cord blood (UCB) units obtained from full-term deliveries were collected from the unborn placenta with the informed consent of the mothers. The human UCB-MSC isolation procedure was approved by the Institutional Review Board of Samsung Medical Center (IRB No. SMC 2019-11-026), and all participants provided informed consent for the use of the umbilical cord in this experimental study. The human monocyte cell line THP-1 was purchased from the Korean Cell Bank (Seoul, Korea) and maintained in complete RPMI 1640 media.

### 2.2. Culture of 3D hUCB-MSC Spheroids

The hUCB-MSCs were grown in minimum essential medium-alpha (MEM-*α*; Gibco, Waltham, MA, USA) supplemented with 10% fetal bovine serum (FBS; Gibco) and 1% penicillin/streptomycin (Gibco) at 37°C and 5% CO_2_ until passage 5. To obtain 3D spheroids, hUCB-MSCs at three to five passages were seeded into round-bottom 96-well plates coated with poly(2-hydroxyethyl methacrylate) (pHEMA; Sigma-Aldrich, St. Louis, MO, USA) reagent prepared in 95% ethanol. In each well containing 100 *μ*L of MEM-*α* (Gibco) supplemented with 10% FBS (Gibco) and 1% penicillin/streptomycin (Gibco), 2.5 × 10^3^, 6.25 × 10^3^, and 25 × 10^3^ MSCs/well were seeded and cultured at 37°C and 5% CO_2_ for three days (2.5 × 10^3^, 6.25 × 10^3^, and 25 × 10^3^ cells/spheroid referred to as 2.5 K, 6.25 K, and 25 K 3D hUCB-MSCs, respectively). The cell numbers were based on the number of seeded cells before spheroid formation. Then, the 3D spheroids were transferred into a Petri dish to obtain conditioned media (CM). Only one spheroid was formed per well of the 96-well plate, and all 96 spheroids were transferred into 15 mL of MEM-*α* (Gibco) supplemented with 10% Exo-free FBS (Gibco) and 1% penicillin/streptomycin (Gibco) in a 100 mm diameter Petri dish. The 3D spheroids were cultured at 37°C and 5% CO_2_ for six days. Every 48 h, 13 mL of conditioned medium was collected and replaced with an equal volume of fresh medium. This resulted in a total of 39 mL of conditioned medium per Petri dish with a total of 96 hUCB-MSC spheroids.

### 2.3. Isolation and Characterization of EVs

Monolayer (2D)-cultured and 3D-cultured hUCB-MSC spheroids were incubated in MEM-*α* (Gibco) supplemented with 10% exosome-depleted FBS (Gibco) for 72 h at 37°C in a fully humidified 5% CO_2_ atmosphere. The CM was harvested and centrifuged at 2,000 × *g* for 10 min to remove cells and debris. Then, the supernatant was concentrated using an Amicon Ultra Centrifugal Filter (100 kDa cut-off) Unit (Merck, Burlington, VT, USA). EVs were isolated from concentrated CM using a Total Exosome Isolation Kit (Invitrogen, Thermo Fisher Scientific, Waltham, MA, USA). The protein in EVs was quantified using a BCA Protein Assay (Thermo Fisher Scientific). The size distribution and concentration of EVs were measured by nanoparticle tracking analysis (NTA) using a NanoSight NS300 (Malvern, Worcestershire, UK). The sample was diluted to a number concentration of between 10^8^ and 10^9^ particles per milliliter. The morphology of the EVs was characterized using transmission electron microscopy (TEM; Hitachi HT7700, Hitachi Ltd., Tokyo, Japan). The EVs were fixed with 4% paraformaldehyde and spotted onto a carbon-coated 300-mesh grid. Then, the samples were stained with 1% uranyl acetate. Subsequently, the samples were examined at 100.0 keV.

### 2.4. Mouse Islet Isolation and Culture

Mouse islets were isolated from 8-12-week-old hIAPP^+/-^ mice as described previously [24]. Briefly, 0.8 mg/mL collagenase P (Roche, Basel, Switzerland) in Hanks' balanced saline solution (HBSS; Sigma-Aldrich) was infused into the common bile duct for mouse pancreas digestion. Islets were purified from the digested pancreas using a Human PanColl (PAN-Biotech GmbH, Am Gewerbepark, Aidenbach, Germany) gradient and washed several times with HBSS (Sigma-Aldrich). Purified hIAPP^+/-^ mouse islets were cultured in RPMI 1640 (Gibco) containing 10,000 U/mL penicillin-streptomycin (Gibco) and cultured at 37°C in a fully humidified 5% CO_2_ atmosphere.

### 2.5. Coculture of Mouse Islets with hUCB-MSCs or hUCB-MSC-Derived EVs

Islets were hand-picked with a microscope at an average size of 150 *μ*m and cultured in serum deprivation conditions (RPMI 1640 supplemented with bovine serum albumin, BSA, 0.625%) for 48 h. During *ex vivo* culture of islets, six experimental groups were designated for experiments using hIAPP^+/-^ FVB/N mouse islets: medium supplemented with 10% FBS (FBS group), medium supplemented with 0.625% BSA (Qbiogene, Carlsbad, CA, USA) (BSA group), medium supplemented with 20 *μ*g/mL 2D hUCB-MSC-derived EVs plus 0.625% BSA (BSA+2D EV group), medium supplemented with 20 *μ*g/mL 25 K 3D hUCB-MSC-derived EVs plus 0.625% BSA (BSA+3D EV group), coculture with 2D hUCB-MSCs in medium supplemented with 0.625% BSA (BSA+2D MSC group), and coculture with 25 K 3D hUCB-MSC spheroids in medium supplemented with 0.625% BSA (BSA+3D MSC group). The numbers of 2D and 3D MSCs per media volume in the BSA+2D MSC and BSA+3D MSC groups were determined as the expected numbers required to yield equivalent amounts of EV per volume in their counterpart groups (BSA+2D EV and BSA+3D EV groups). For coculture of islets with 2D MSCs, 5 × 10^5^ MSCs were seeded in six-well plates overnight before the islets were added. For coculture of islets with 25 K 3D spheroids, 28 spheroids were transferred to a low-attachment six-well plate. From each well, 50 islets were hand-picked onto a cell culture insert (Falcon, Corning, NY, USA) and incubated for 48 h at 37°C in a fully humidified 5% CO_2_ atmosphere.

### 2.6. Coculture of Mouse Islets with Pancreatic Macrophages with or without MSC-Derived EVs

For coculture of islets with pancreatic macrophages, 1 × 10^6^ isolated pancreatic macrophages were seeded in six-well plates and incubated for 2 h with complete RPMI 1640 media. Nonadherent cells were removed, and 50 islets were seeded above the cell culture insert with fresh complete RPMI 1640 media. Islets were cocultured only with macrophages (control group), 10 *μ*g/mL 2D MSC EV treatment (2D hUCB-MSC-derived EV group), or 10 *μ*g/mL 3D MSC EV treatment (3D hUCB-MSC-derived EV group). All samples were cultured in complete RPMI1640 media for 48 h.

### 2.7. Pancreatic Macrophage Isolation

To isolate pancreatic macrophages, STZ-induced type 1 diabetes was established by s.c. administration of STZ at the dose of 120 mg/kg body weight. At 4-day post-STZ injection, pancreas tissue was digested in Hanks balanced saline solution (Sigma-Aldrich) containing 2 mg/mL of collagenase P (Roche) for 15 min at 37°C. Digestion was quenched by FBS, and the digestate was filtered through 70 *μ*m Nylon mesh and centrifuged at 350 g for10 min. Cell suspensions were resuspended, and anti-F4/80 microbeads (Miltenyi Biotec, Bergisch Gladbach, Germany) were used for negative selection of macrophages according to the manufacturer's instructions.

### 2.8. Real-Time Reverse Transcription-PCR

Total RNA was isolated from mouse islets using an RNeasy Mini Kit (Qiagen, Hilden, Germany) according to the manufacturer's protocol. RNA (1 *μ*g) was reverse-transcribed to obtain cDNA using PrimeScript RT Master Mix (Takara Bio Inc., Kusatsu, Japan). Real-time quantitative reverse transcription- (RT-) PCR was performed in triplicate using gene-specific primers. PCR was performed with a TB Green Premix Kit (Takara) and 7500 Fast Real-Time PCR System (Applied Biosystems, Waltham, MA, USA). The relative gene expression of TNF-*α*, interleukin- (IL-) 1 beta (1*β*), IL-18, NOD-like receptor pyrin domain-containing protein 3 (NLRP3), high mobility group box 1 (HMGB1), caspase-1, Oct4, neurogenin 3 (NGN3), forkhead box protein O1 (FoxO1), and pancreatic and duodenal homeobox 1 (Pdx1) was determined and normalized to that of mouse *β*-actin using gene-specific primer pairs (see Supplementary Materials, Table 1).

For the microRNA real-time PCR assay, total RNA was extracted from 30 *μ*g EVs using a Total Exosome RNA and Protein Isolation Kit (Thermo Fisher Scientific). The relative expression (ΔΔCt) of microRNA suggested to induce polarization of macrophages into M1 (miR-127-3p and miR-155-5p) and M2 (miR-34a-5p and miR-146a-5p) phenotypes which were determined using a Mir-X™ miRNA qRT-PCR TB Green® Kit (Takara), according to manufacturer's instructions. These microRNAs were chosen based on a previous study that used exosomes from proinflammatory cytokine-stimulated adipose MSCs [20]. The entire sequence of the mature microRNA can be used as the microRNA-specific 5′ primer.

### 2.9. Western Blot Analysis

For protein extraction, hUCB-MSC-derived EVs were lysed with RIPA buffer. Equal amounts of proteins from each group were separated by 10% sodium dodecyl sulfate (SDS) PAGE and transferred to a PVDF membrane. Anti-CD63 (System Biosciences, LLC, Palo Alto, CA, USA), anti-TSG101 (System Biosciences), anti-CANX (ABclonal, Woburn, MA, USA), and anti-GM130 (ABclonal) were used as primary antibodies. Horseradish peroxidase- (HRP-) conjugated IgG antibody (ABclonal) was used as the secondary antibody. Antibody-bound proteins were visualized using an enhanced chemiluminescent reagent.

### 2.10. Glucose-Induced Insulin Secretion (GSIS) Test

A GSIS test was performed to measure islet functionality as described previously [24]. Islets cultured with or without hUCB-MSC-derived EVs were cultured in a KRBB solution containing low- (60 mg/dL) or high-concentration glucose (300 mg/dL). Twenty islets were used for each condition. After incubating for 60 min at 37°C, the supernatant was collected for measuring insulin concentration by ultrasensitive insulin enzyme-linked immunosorbent assay (ELISA) (ALPCO, Salem, NH, USA).

### 2.11. Enzyme-Linked Immunosorbent Assay and Cytokine Array

To determine the expression level of cytokines, mouse islets (80 islets per well) were seeded onto a 6-well ultralow attachment plate (Corning, NY, USA). The culture medium was collected at 48 h without cellular debris. The concentrations of TNF-*α*, IL-1*β*, IL-6, and HMGB1 were quantified with an ELISA kit (R&D Systems, Minneapolis, MN, USA) according to the manufacturer's protocol. A cytokine array was performed using a Proteome Profiler Human XL Cytokine Array Kit (R&D Systems) following the manufacturer's instructions.

### 2.12. Cytokine Profiling Array

A cytokine array was performed using a Proteome Profiler Human XL Cytokine Array Kit (R&D Systems) following the manufacturer's instructions. For cytokine arrays, the membrane was blocked in buffer and incubated with 100 *μ*g of EVs overnight at 4°C on a rocking platform shaker. Then, the membrane was incubated with a detection antibody cocktail for 1 h on a shaker. After washing, the membrane was incubated with streptavidin-HRP. Finally, Chemi Reagent mix was spread on the membrane, and an autoradiograph was obtained. Pixel densities were analyzed using NIH ImageJ medical imaging software.

### 2.13. Flow Cytometry Analysis

Cells were washed and resuspended in staining buffer. Fluorochrome-conjugated monoclonal antibodies were added to cells and incubated for 30 min in the dark. An hUCB-MSC surface marker analysis with flow cytometry was performed using the following monoclonal antibodies: CD105, CD73, CD90, CD45, CD34, CD14, CD11b, CD19, HLA-DR, positive isotype control cocktail (mIgG1 FITC, mIgG1 PerCP-Cy5.5, and mIgG1 APC), and negative isotype control cocktail (mIgG1 PE and mIgG2a PE) purchased from BD Biosciences (San Jose, CA, USA). Phenotyping of macrophages with flow cytometry was performed using the antibodies of M2 macrophage differentiation markers (CD163, BD Biosciences; CD206, Abcam, Cambridge, UK) and M1 macrophage differentiation markers (CD80; Abcam). After staining of primary antibody, cells were washed and resuspended in 150 *μ*L of the appropriate secondary antibody (Alexa Fluor® 488-conjugated donkey anti-rat, Alexa Fluor® 647-conjugated donkey anti-rabbit; Abcam). Data were acquired using BD LSRFortessa flow cytometers and analyzed with BD FACSDiva software (BD Biosciences, Franklin Lakes, NJ, USA).

### 2.14. Fluorescence Immunohistochemistry

Islets were harvested, fixed in 4% paraformaldehyde, and immersed in 30% sucrose solution to be embedded in a frozen block. Inflammatory markers and dedifferentiation markers were detected in cell sections by immunofluorescence. Anti-FoxO1 (Santa Cruz, Dallas, Texas, USA), anti-cleaved caspase 3 (Cell Signaling, Danvers, MA, USA), anti-Oct4 (Abcam, Cambridge, UK), anti-NGN3 (Abcam), anti-A11 oligomer (Invitrogen), anti-Pdx1 (Abcam), anti-IL-1*β* (Abcam), and anti-insulin (Abcam) were used as primary antibodies. M1/M2 macrophage markers were identified using the antibodies of M2 macrophage differentiation markers (anti-arginase-1; Cell Signaling) and M1 macrophage differentiation markers (CD80; Abcam). Sections were incubated with primary antibody diluted in blocking buffer overnight at 4°C. Secondary antibodies (Alexa Fluor® 488-conjugated goat anti-rabbit, Alexa Fluor® 488-conjugated goat anti-mouse, and Alexa Fluor® 594-conjugated goat anti-rabbit; Abcam) were added and incubated for 1 h at room temperature. Counterstaining was performed using DAPI (1 : 10,000). Images were obtained from each section using a fluorescent microscope (Olympus, Shinjuku, Tokyo, Japan).

### 2.15. Statistical Methods

Results are reported as mean ± standard deviation. Statistical analysis was performed using GraphPad Software (GraphPad, San Diego, CA, USA). Continuous variables were compared using one-way analysis of variance (ANOVA) or the Mann–Whitney *U* test, as appropriate. *P* values < 0.05 were considered significant.

## 3. Results and Discussion

### 3.1. Characterization of EVs Derived from Umbilical Cord Blood MSCs

**Figure 1:**
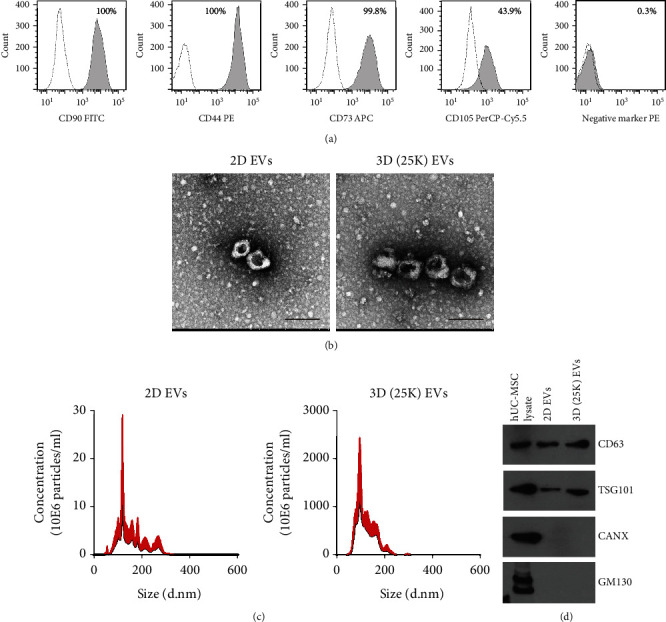
Characterization of 2D and 3D hUCB-MSC-derived extracellular vesicles (EVs). (a) MSC markers, CD90, CD73, CD44, and CD105, and MSC negative markers were analyzed by flow cytometry. The white curve represents the isotype control, and the light gray curve represents stained cells. (b) 2D and 3D hUCB-MSCB-derived EVs were analyzed by transmission electron microscopy (TEM). Scale bar = 200 nm. (c) The size distribution of 2D and 3D hUCB-MSC-derived EVs were analyzed by nanoparticle tracking analysis (NTA). (d) EV markers CD63 and TSG101 were identified by western blotting.

To confirm the identity of the isolated hUCB-MSCs, surface expression of CD90, CD44, CD73, and CD105 and the nonexpression of the negative marker cocktail (CD45, CD34, CD14, CD11b, CD19, and HLA-DR) were confirmed using flow cytometry (Figure 1(a)). After conditioned medium was obtained during 2D and 3D cultures of hUCB-MSCs, hUCB-MSC-derived EVs were isolated from each group using a polymer-based precipitation method. The shape of each EV was observed using TEM. The cup-shaped morphology of the isolated EVs was confirmed in both 2D and 3D hUCB-MSC-derived EVs (Figure 1(b)). There was no significant change in the size distribution of EVs according to the MSC culture method (Figure 1(c)). When the expression of EV markers was examined using western blotting, CD63 and TSG101 were expressed, and the ER and Golgi markers CANX and GM130 were not expressed (Figure 1(d) and Supplementary Figure S1).

### 3.2. hUCB-MSC-Derived EVs Promote M2 Polarization in Pancreatic Macrophages and THP-1 Monocytes

**Figure 2:**
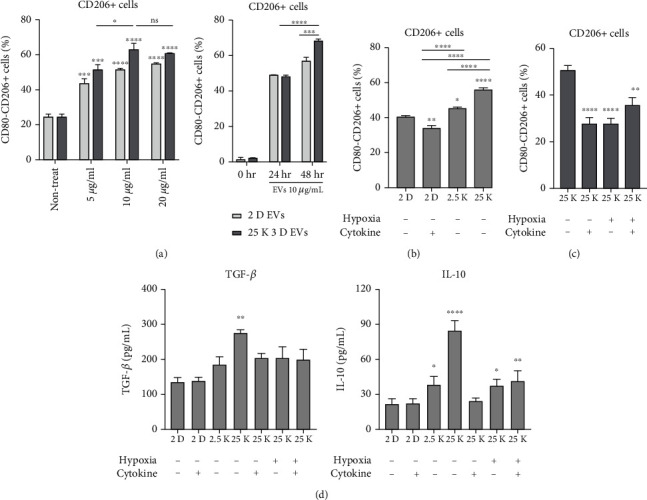
3D hUCB-MSC-derived extracellular vesicles (EVs) and M2 polarization of pancreatic macrophages. (a) Flow cytometry analysis of cell surface molecules CD206 and CD80 on pancreatic macrophages cultured with monolayer (2D) and 25 K 3D hUCB-MSC-derived EVs. The error bars represent the standard deviation of measurements in three separate sample runs (*n* = 6). Significance of difference was determined using two-way analysis of variance (ANOVA) with Tukey's posttest. (b) Flow cytometry analysis of cell surface molecules CD206 and CD80 on macrophages cultured in the presence of EVs isolated from the supernatants of 2D- or 3D-cultured (2.5 K and 25 K) hUCB-MSCs. The error bars represent the standard deviation of measurements in three separate sample runs (*n* = 6). (c) Flow cytometry analysis of cell surface molecules CD206 and CD80 on pancreatic macrophages cultured in the presence of EVs isolated from the supernatants of hUCB-MSCs that were unstimulated or preconditioned with hypoxia or cytokines (TNF-*α* and IFN-*γ*, each 40 ng/mL). The error bars represent the standard deviation of measurements in three separate sample runs (*n* = 6). (d) Medium concentrations of TGF-*β* and IL-10 were measured by ELISA. The error bars represent the standard deviation of measurements in three separate sample runs (*n* = 8). Significance of difference was determined using one-way ANOVA with Tukey's posttest. Columns, mean; bars, SD. ^∗^*p* < 0.05, ^∗∗^*p* < 0.005, ^∗∗∗^*p* < 0.0005, and ^∗∗∗∗^*p* < 0.0001 compared to the control group.

Next, we evaluated whether hUCB-MSC-derived EVs increase M2 polarization in isolated pancreatic macrophages from FVB mice. The expression of an M2 polarization marker (CD206) in pancreatic macrophage cells increased after treatment with 2D and 3D hUCB-MSC-derived EVs for 48 h. The results showed that hUCB-MSC-derived EVs could promote M2 polarization of macrophages in a concentration-dependent manner, showing 10 *μ*g/mL as the likely optimal concentration (Figure 2(a)). To determine the M2 polarization activity of EVs, 2D and different sizes of 3D hUCB-MSC-derived EVs (10 *μ*g/mL) were added to cultures of isolated macrophages, and the expression of M2 polarization markers (CD206) was identified using flow cytometry (Figure 2(b) and Supplementary Figure S2a). Compared to the macrophages treated with 2D hUCB-MSC-derived EVs with and without cytokines (IFN-*γ* and TNF-*α*, each 40 ng/mL), the M2 polarization marker CD206 was upregulated in the macrophages treated with 3D hUCB-MSC-derived EVs. When the macrophages were treated with 3D hUCB-MSC-derived EVs, the expression of CD206 was higher when the seeding density of hUCB-MSC was 25 K cells per spheroid than for 2.5 K cells per spheroid. When macrophages were treated with 3D hUCB-MSC-derived EVs, the expression of CD206 was significantly higher in macrophages cultured with 25 K 3D hUCB-MSC-derived EVs than with 2.5 K 3D hUCB-MSC-derived EVs (Figure 2(b)). Consistent findings were observed when M2 polarization markers (CD206 and CD163) of THP-1 monocytes were compared among groups (Supplementary Figure S3).

To evaluate whether the additional preconditioning of hUCB-MSCs, such as exposure to hypoxia and cytokine, further potentiates the M2 polarization ability of EVs, we obtained EVs from 3D cultures of hUCB-MSCs grown under hypoxia or cytokines (TNF-*α* and IFN-*γ*, each 40 ng/mL) at a seeding density of 25 × 10^3^ cells per spheroid (Figure 2(c) and Supplementary Figure S2b). Preconditioning with cytokines did not further increase the M2 polarization ability of 3D hUCB-MSC-derived EVs. When cytokine secretion of the macrophages cultured in the presence of 2D and 3D hUCB-MSC-derived EVs was measured by ELISA, the IL-10 and TGF-*β* levels were significantly increased in macrophages cultured in the presence of 25 K 3D hUCB-MSC-derived EVs (Figure 2(d)).

**Figure 3:**
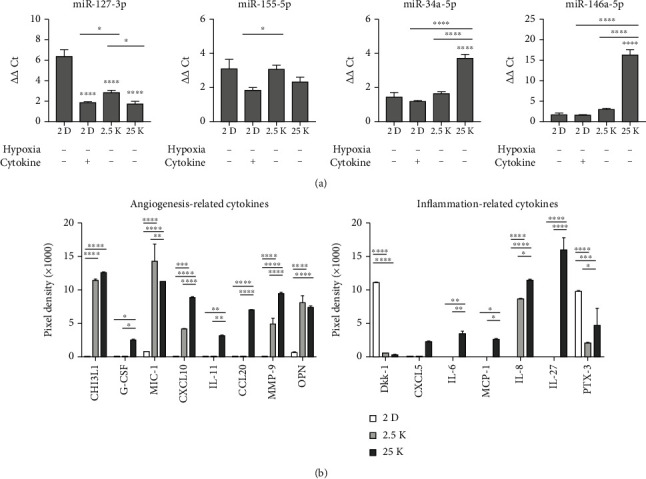
The cargo of hUCB-MSC-derived EVs from each condition. (a) The levels of miR-127-3p, miR-155-5p, miR-34a-5p, and miR-146a-5p were measured by qRT-PCR in EVs derived from unstimulated 2D, cytokine (IFN-*γ* and TNF-*α*, each 40 ng/mL)-stimulated 2D, and 3D hUCB-MSCs. The error bars represent the standard deviation of measurements in three separate sample runs (*n* = 10). Significance of difference was determined using one-way ANOVA with Tukey's posttest. (b) Proteomic analysis of selected human cytokines from 2D and 3D hUCB-MSC-derived EVs. The error bars represent the standard deviation of measurements in two separate sample runs (*n* = 4). Significance of difference was determined using two-way ANOVA with Tukey's posttest. Columns, mean; bars, SD. ^∗^*p* < 0.05, ^∗∗^*p* < 0.005, ^∗∗∗^*p* < 0.0005, and ^∗∗∗∗^*p* < 0.0001.

A recent study suggested that EVs from cytokine-preconditioned MSCs contain several miRs that can induce macrophage polarization into the M2 subtype, in contrast to the EVs from resting MSCs [20, 21]. Thus, miRs in EVs isolated after preconditioning with IFN-*γ* and TNF-*α* in 2D- and 3D-cultured hUCB-MSC-derived EVs were quantified and compared with miRs in EVs isolated from 2D cultures without cytokine stimulation. When the cargoes of 2D and 3D (25 K) hUCB-MSC-derived EVs were analyzed, the level of miR-127-3p, a miR involved in M1 polarization [20], was decreased in 25 K 3D hUCB-MSC-derived EVs compared to 2.5 K and 2D hUCB-MSC EVs, resulting in a similar level to that in cytokine-treated 2D hUCB-MSC EVs (Figure 3(a)). There was no difference in the level of miR-155-5p between 25 K 3D hUCB-MSC-derived EVs and 2D hUCB-MSC-derived EVs. The level of miR-155-5p was increased in the 2.5 K 3D hUCB-MSC EVs (Figure 3(a)). In 25 K 3D hUCB-MSC-derived EVs, the levels of miR-34a-5p and miR-146a-5p, miRs involved in M2 polarization [20], were significantly greater than those in unstimulated 2D, cytokine-stimulated 2D, and other 3D hUCB-MSC-derived EVs. A significantly higher level of miR-34a-5p was observed in 2.5 K 3D hUCB-MSC-derived EVs than in unstimulated and cytokine-stimulated 2D hUCB-MSC-derived EVs (Figure 3(a)). Based on these results, 25 K 3D hUCB-MSC-derived EVs without exposure to hypoxia and cytokines were used for further experiments.

A comparison of the protein expression profiles of 2D MSC-derived EVs and 3D MSC-derived EVs revealed that cytokines related to angiogenesis and inflammation, such as IL-6, MCP-1, MIC-1, IL-11, G-CSF, CCL20, and IL-27, were expressed in higher quantities in 3D MSC-derived EVs than in 2D MSC-derived EVs (Figure 3(b)).

### 3.3. hUCB-MSC-Derived EVs Attenuate Transcription of Proinflammatory Cytokines in hIAPP^+/-^ Mouse Pancreatic Islets during Serum-Deprived Culture

**Figure 4:**
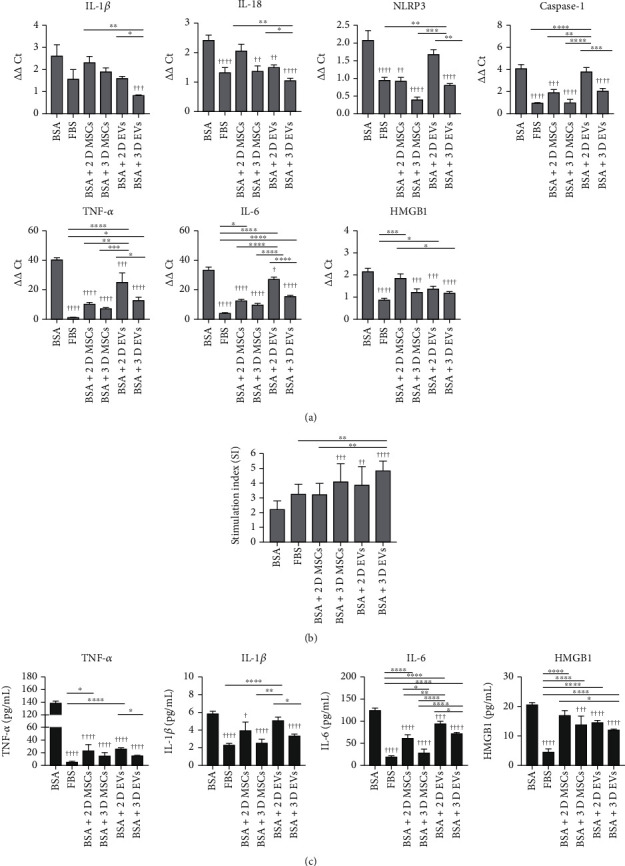
Effects of 3D hUCB-MSC-derived EVs on the expression of cytokines and insulin secretion of islets. Islets isolated from hIAPP^+/-^ mice were cultured under four conditions: media supplemented with fetal bovine serum (FBS; FBS group), media supplemented with bovine serum albumin (BSA) without FBS (BSA group), media supplemented with BSA without FBS but with 2D hUCB-MSC (BSA+2D EV group), and media supplemented with BSA without FBS but with 3D hUCB-MSC (BSA+3D EV group). (a) Concentrations of IL-1*β*, IL-18, NLRP3 inflammasome, caspase-1, TNF-*α*, IL-6, and HMGB1 in hIAPP^+/-^ islet cells treated with EV produced by 2D- or 3D-cultured hUCB-MSCs were measured using qRT-PCR. The error bars represent the standard deviation of measurements in three separate sample runs (*n* = 9). (b) Glucose-stimulated insulin secretion (GSIS) of hIAPP^+/-^ mouse islets in each group. GSIS was evaluated using ELISA at 48 h after statin incubation of islets in low (60 mg/dL) and high (300 mg/dL) glucose. The error bars represent the standard deviation of measurements in three separate sample runs (*n* = 10). (c) Medium concentrations of TNF-*α*, IL-6, IL-1*β*, and HMGB1 were measured by ELISA. The error bars represent the standard deviation of measurements in four separate sample runs (*n* = 12). Columns, mean; bars, SD. ^∗^*p* < 0.05, ^∗∗^*p* < 0.005, ^∗∗∗^*p* < 0.0005, and ^∗∗∗∗^*p* < 0.0001. ^†^*p* < 0.05, ^††^*p* < 0.005, ^†††^*p* < 0.0005, and ^††††^*p* < 0.0001 compared to the BSA group by one-way ANOVA and Tukey's posttest.

To examine whether hUCB-MSC-derived EVs could inhibit nonspecific inflammation and *β*-cell dysfunction provoked by serum deprivation, islets isolated from hIAPP^+/-^ mice were cultured in media supplemented with BSA without FBS (BSA group), media supplemented with FBS (FBS group), and media supplemented with BSA without FBS but with EVs derived from 2D hUCB-MSCs (BSA+2D EV group) or 3D hUCB-MSCs (BSA+3D EV group) or cocultured with 2D hUCB-MSCs (BSA+2D MSC group) or 3D hUCB-MSCs (BSA+3D MSC group) in media supplemented with BSA without FBS. The mRNA transcription levels of IL-1*β*, IL-18, NLRP3, caspase-1, TNF-*α*, IL-6, and HMGB1 in the six groups were measured using quantitative real-time PCR (Figure 4(a)). The transcription levels of IL-1*β*, IL-18, NLRP3, caspase-1, TNF-*α*, IL-6, and HMGB1 were all significantly lower in the BSA+3D EV group than in the BSA group. The transcription levels of TNF-*α*, IL-18, IL-6, and HMGB1 but not IL-1*β*, NLRP3, and caspase-1 were also significantly lower in the BSA+2D EV group than in the BSA group. The transcription levels of TNF-*α*, IL-1*β*, IL-18, NLRP3, IL-6, and caspase-1 were lower in the BSA+3D EV group than in the BSA+2D EV group (Figure 4(a)). We then evaluated the effect of hUCB-MSC-derived EVs on GSIS in hIAPP^+/-^ islets, as shown in Figure 4(b). High-glucose (300 mg/dL)-stimulated insulin secretion islet cells from 3D hUCB-MSC-derived EVs over a one-hour static incubation period were more numerous than in the BSA group.

At least in part, the decrease in the transcription levels of IL-18, NLRP3, caspase-1, TNF-*α*, IL-6, and HMGB1 in the BSA+3D MSC group was reproducible in the BSA+3D EV group. The decrease in medium concentrations of TNF-*α*, IL-1*β*, and HMGB1 in the BSA+3D MSC group and the BSA+3D EV group was comparable, and the decrease in medium concentration of IL-6 in the BSA+3D MSC group was partly reproducible in the BSA+3D EV group (Figure 4(c)). The decrease in transcription levels of TNF-*α* and IL-6 in the BSA+2D MSC group was partly reproducible in the BSA+2D EV group. The decrease in medium concentration of TNF-*α* and in part of the IL-6 in the BSA+2D MSC group was reproducible in the BSA+2D EV group. The decrease in medium concentration of IL-1*β* in the BSA+2D MSC group was not reproducible in the BSA+2D EV group (Figure 4(c)).

**Figure 5:**
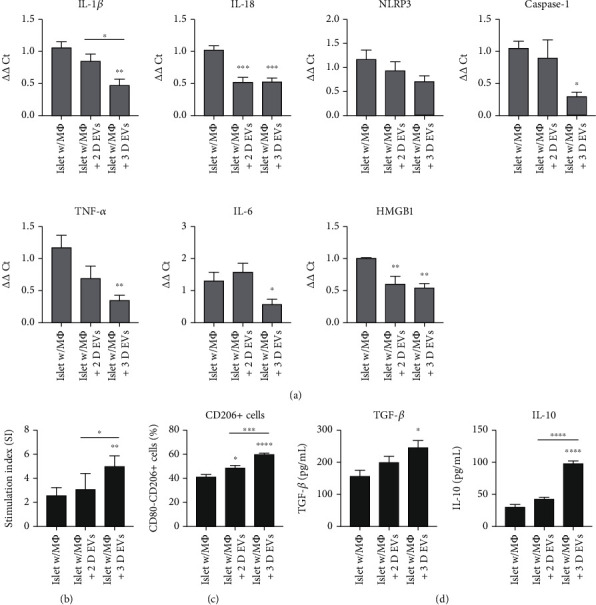
M2 polarization ability of hUCB-MSC-derived EVs in a coculture system of islets and pancreatic macrophages. In a transwell system, 50 islets and 1 × 10^6^ macrophages were cultured under three conditions: islets with macrophages without hUCB-MSC-derived EVs (islet w/M*Φ*), islets with macrophages with 2D hUCB-MSC-derived EVs (islet w/M*Φ*+2D EVs), and islets with macrophages with 3D hUCB-MSC-derived EVs (islet w/M*Φ*+3D EVs). (a) Concentrations of IL-1*β*, IL-18, NLRP3 inflammasome, caspase-1, TNF-*α*, IL-6, and HMGB1in cocultured hIAPP*^+/-^* islet cells treated with EV produced by 2D- or 3D-cultured hUCB-MSCs were measured using qRT-PCR. The error bars represent the standard deviation of measurements in three separate sample runs (*n* = 9). (b) Glucose-stimulated insulin secretion (GSIS) of hIAPP*^+/-^* mouse islets in each group. GSIS was evaluated using ELISA at 48 h after statin incubation of islets in low (60 mg/dL) and high (300 mg/dL) glucose. The error bars represent the standard deviation of measurements in three separate sample runs (*n* = 6). (c) Flow cytometry analysis of cell surface molecules CD206 and CD80 on macrophages cultured in the presence of EVs. The error bars represent the standard deviation of measurements in three separate sample runs (*n* = 9). (d) Medium concentrations of TGF-*β* and IL-10 were measured by ELISA. The error bars represent the standard deviation of measurements in four separate sample runs (*n* = 9). Columns, mean; bars, SD. ^∗^*p* < 0.05, ^∗∗^*p* < 0.005, ^∗∗∗^*p* < 0.0005, and ^∗∗∗∗^*p* < 0.0001 by one-way ANOVA and Tukey's posttest.

To determine whether these findings are consistent in the setting of interaction among pancreatic islets, macrophages, and EVs from MSCs, we cocultured pancreatic islets from hIAPP^+/-^ mice and freshly isolated pancreatic macrophages using a transwell system, with or without medium supplementation of 2D or 3D hUCB-MSC-derived EVs. After 48 hours of coculture, the total RNA of the pancreatic islets was extracted and real-time RT-PCR was performed. The transcription levels of IL-1*β*, IL-18, caspase-1, TNF-*α*, IL-6, and HMGB1 were downregulated in the islets cocultured with pancreatic macrophages in the presence of 3D hUCB-MSC-derived EVs (Figure 5(a)). High-glucose (300 mg/dL)-stimulated insulin secretion over a 1 h static incubation period in islet cells from 3D hUCB-MSC-derived EVs was greater than in the control group or the 2D hUCB-MSC-derived EV group (Figure 5(b)). To evaluate the hUCB-MSC-derived EV-mediated M2 polarization, cocultured macrophages were stained with CD80 and CD206. The proportion of CD80-CD206+ cells was significantly greater in the 3D hUCB-MSC-derived EV groups than in the control and 2D hUCB-MSC-derived EV groups, suggesting that 3D hUCB-MSC-derived EVs promoted M2 polarization of macrophages in the setting of interaction among pancreatic islets, macrophages, and EVs from MSCs (Figure 5(c)). When the concentrations of TGF-*β* and IL-10 in the supernatant were determined by ELISAs in this setting, both significantly increased in the presence of 3D hUCB-MSC-derived EVs compared to the control group. The concentration of IL-10 in the 3D hUCB-MSC derived EV group was significantly higher than that of the 2D hUCB-MSC derived EV group (Figure 5(d)).

**Figure 6:**
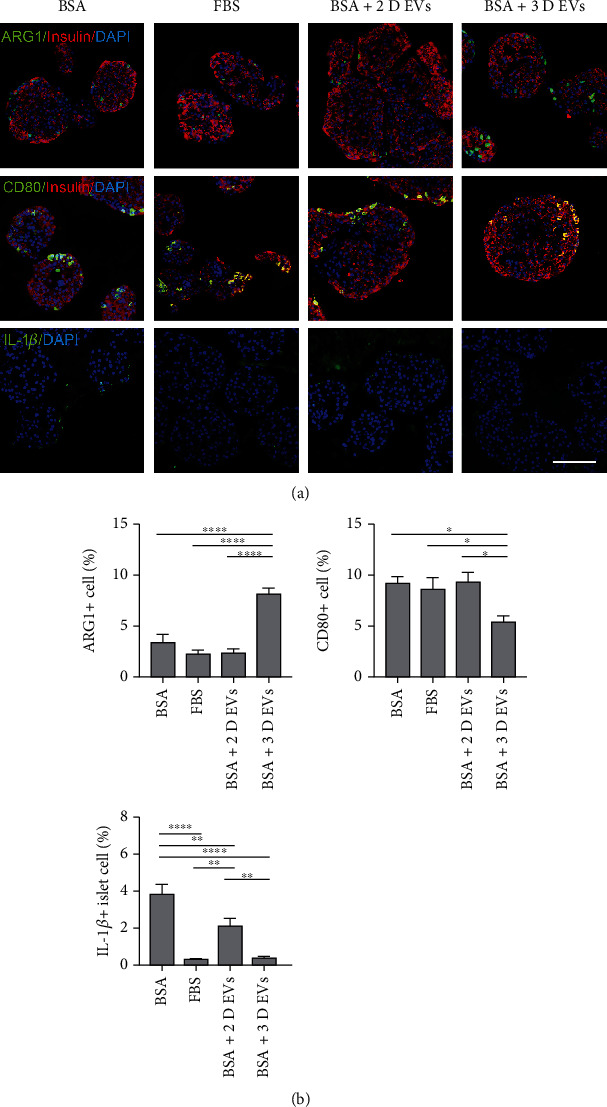
Effect of 3D hUCB-MSC-derived EVs on M1/M2 polarization of islet-resident macrophages in islets. The hIAPP^+/-^ mouse islets were cultured under four conditions: medium supplemented with 10% fetal bovine serum (FBS group), medium supplemented with 0.625% bovine serum albumin (BSA group), 20 *μ*g/mL 2D hUCB-MSC-derived EVs plus 0.625% BSA (BSA+2D EV group), and 20 *μ*g/mL 3D hUCB-MSC-derived EVs plus 0.625% BSA (BSA+3D EV group). (a) Immunocytochemical staining with antibodies against insulin (red), DAPI (blue), and ARG1, CD80 and IL-1*β* (green). Scale bar = 100 *μ*m. (b) The percentage proportions of ARG1-, CD80-, and IL-1*β*-stained cells (green) among total DAPI-positive islet cells (blue; 50~100 islets per group). The error bars represent the standard deviation of measurements in three separate sample runs (*n* = 12). Columns, mean; bars, SD. ^∗^*p* < 0.05, ^∗∗^*p* < 0.005, ^∗∗∗^*p* < 0.0005, and ^∗∗∗∗^*p* < 0.0001 by one-way ANOVA and Tukey's posttest.

To confirm whether the inhibition of nonspecific inflammation caused by EVs derived from hUCB-MSCs was associated with the M1/M2 polarization of islet-resident macrophages, the proportion of ARG1^+^ and CD80^+^ cells in the islets of each group was compared. Although the proportion of ARG1^+^ cells in the islets of the BSA+2D EV group was not different from that of the BSA and FBS groups, the proportion of ARG1^+^ cells in the islets of the BSA+3D EV group was higher than in the other groups. Although the proportion of CD80^+^ cells in the islets of the BSA+2D EV group was not different from that of the FBS group, the proportion of ARG1^+^ cells in the islets of the BSA+3D EV group was lower than in the BSA, FBS, and BSA+2D EV groups (Figures 6(a) and 6(b)). The proportion of IL-1*β*^+^ cells in the islets of both the BSA+3D EV and BSA+2D EV groups was lower than that of the BSA group. A more potent reduction in the proportion of IL-1*β*^+^ cells in islets was observed in the BSA+3D EV group, which was similar to that of the FBS group (Figures 6(a) and 6(b)).

### 3.4. hUCB-MSC-Derived EVs Attenuate Dedifferentiation of hIAPP^+/-^ Mouse Pancreatic Islets during Serum-Deprived Culture

**Figure 7:**
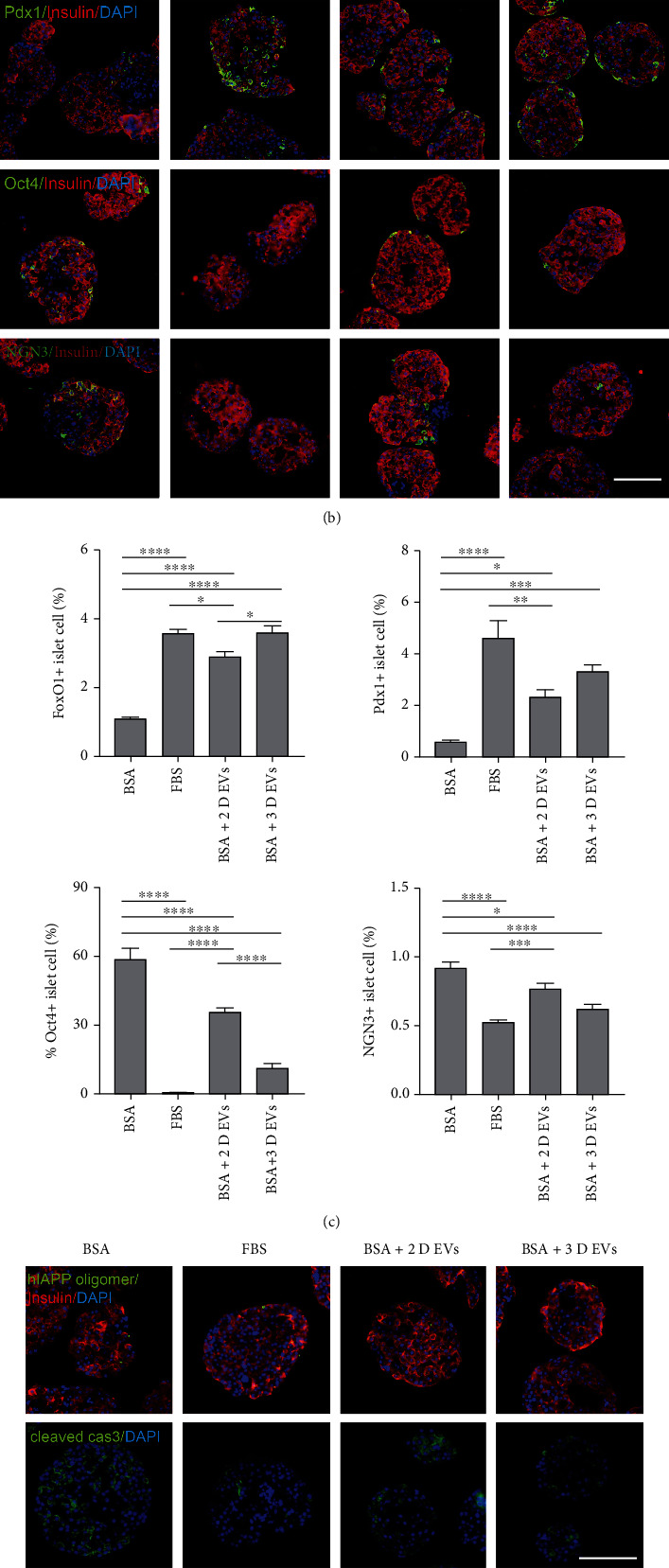
Effect of 3D hUCB-MSC-derived EVs on the expression of *β*-cell identity markers in islets. The hIAPP^+/-^ mouse islets were cultured under four conditions: medium supplemented with 10% fetal bovine serum (FBS group), medium supplemented with 0.625% bovine serum albumin (BSA group), 20 *μ*g/mL 2D hUCB-MSC-derived EVs plus 0.625% BSA (BSA +2D EV group), and 20 *μ*g/mL 3D hUCB-MSC-derived EVs plus 0.625% BSA (BSA +3D EV group). (a) Concentrations of Oct4, NGN3, Pdx1, and FoxO1 in hIAPP^+/-^ islet cells treated with EVs produced by 2D- or 3D-cultured hUCB-MSCs were measured using qRT-PCR. The error bars represent the standard deviation of measurements in three separate sample runs (*n* = 9). (b) Immunocytochemical staining with antibodies against insulin (red); DAPI (blue); and Oct4, NGN3, Pdx1, and FoxO1 (green). Scale bar = 100 *μ*m. (c) The percentage proportions of Oct4-, NGN3-, Pdx1-, and FoxO1-stained cells (green) among total DAPI-positive islet cells (blue; 50~100 islets per group). The error bars represent the standard deviation of measurements in three separate runs (*n* = 12). (d) Immunocytochemical staining with antibodies against insulin (red), DAPI (blue), and hIAPP oligomer and cleaved caspase-3 (green). Scale bar = 100 *μ*m. (e) The percentage proportions of hIAPP oligomer and cleaved caspase-3-stained cells (green) among total DAPI-positive islet cells (blue; 50~100 islets per group). The error bars represent the standard deviation of measurements in three separate sample runs (*n* = 12). Columns, mean; bars, SD. ^∗^*p* < 0.05, ^∗∗^*p* < 0.005, ^∗∗∗^*p* < 0.0005, and ^∗∗∗∗^*p* < 0.0001, by one-way ANOVA and Tukey's posttest.

Metabolic stress-induced proinflammatory cytokines can induce the dedifferentiation of islet cells [25, 26]. Therefore, the mRNA transcription levels of *β*-cell identity markers were compared among the four groups. The mRNA transcription levels of Oct4 and NGN3 in the BSA+2D EV and BSA+3D EV groups were significantly lower than those of the BSA group. In the BSA+3D EV group but not in the BSA+2D EV group, the mRNA transcription levels of Pdx1 and FoxO1 were significantly higher than those in the BSA group, with a significantly higher level in the BSA+3D EV group than in the BSA+2D EV group (Figure 7(a)). The protein expression of *β*-cell identity markers was evaluated using immunocytochemical staining. Similar to real-time RT-PCR assays, immunostaining showed that the expression levels of Pdx1 and FoxO1 were significantly higher in the BSA+3D EV group than in the BSA group. The proportion of FoxO1-positive cells was ~3-fold higher in both the EVs (20 *μ*g/mL) derived from the 3D hUCB-MSC and 2D hUCB-MSC groups than in the BSA group. The proportion of islet cells expressing Oct4 and NGN3 was downregulated in FBS, 2D hUCB-MSC-derived EVs, and 3D hUCB-MSC-derived EVs. The proportion of islets expressing Oct4 was lower in the 3D hUCB-MSC-derived EVs than in the 2D hUCB-MSC-derived EVs.

In addition to alleviating the dedifferentiation of islets, the proportion of islets expressing cleaved caspase-3 was significantly lower in both the 3D hUCB-MSC-derived EV and 2D hUCB-MSC-derived EV groups than in the BSA group (Figures 7(d) and 7(e)), whereas there were no significant differences in the numbers of hIAPP oligomer-positive cells between the BSA and 2D or 3D hUCB-MSC-derived EV groups.

## 4. Discussion and Conclusions

In this study, miRs involved in the M2 polarization of macrophages were more abundant in EVs derived from 3D hUCB-MSCs than in 2D hUCB-MSC- and 3D hUCB-MSC-derived EVs that possessed an enhanced M2 polarization ability on THP-1 monocytes. The protective effects of the 3D hUCB-MSC-derived EVs on hIAPP heterozygote transgenic mouse islets were more potent than 2D hUCB-MSC-derived EVs in terms of reducing nonspecific inflammation and preserving *β*-cell identity, and this was associated with a higher proportion of islet-resident macrophages with M2 polarization markers. To the best of our knowledge, these results are the first evidence that 3D hUCB-MSC-derived EVs have a direct protective effect on hIAPP-expressing pancreatic islets, and the M2-polarizing ability of the EVs might be an important contributor to such protective effects.

The greater M2-polarizing ability of EVs derived from 3D hUCB-MSCs compared to those derived from 2D hUCB-MSCs is a novel finding of this study. Although a recent study revealed that human adipose mesenchymal stem cell- (AMSC-) derived EVs can directly induce the M2 polarization of macrophages through the direct effect of miRs related to M2 polarization, such as that of miR34a-5p and miR 146a-5p [20], it has not been determined if the levels of such miRs can be amplified in 3D cultures of hUCB-MSCs. In the optimized 3D culture conditions in this study, miR34a-5p and miR 146a-5p were more abundant in EVs derived from 3D hUCB-MSCs than in those derived from 2D hUCB-MSCs, even when the 2D MSCs were preconditioned by IFN-*γ* and TNF-*α*. In addition, miR127-3p was less abundant in 3D than in 2D hUCB-MSC-derived EVs. miR-34 inhibits the transcription of proinflammatory cytokines by targeting Notch1, and miR-146 targets NF-*κ*B signaling mediators such as IRAK1 and TRAF6 to promote the expression of M2-associated genes [20, 27, 28]. These results would be relevant to the enhanced protective effects of 3D MSC-derived EVs on *β*-cells because macrophages are associated with inflammation in islet cells and *β*-cell dysfunction [7]. The injection of hUCB-MSCs into a type 2 diabetes mouse model polarized M1 macrophages to M2 macrophages in islets [3], reduced apoptosis of *β*-cells, and increased PDX-1 and MafA expressions. The results of our study indicate that similar effects can be reproduced by 3D hUCB-MSC-derived EVs alone, without the help of cellular components.

In addition to comparing miRs related to M2 polarization, we compared the protein expression profiles of 2D and 3D hUCB-MSC-derived EVs. Proteomic analysis of 3D hUCB-MSC-derived EVs (Figure 3(b)) demonstrated markedly upregulated expression of CHI3L1, IL-6, monocyte chemoattractant protein-1 (MCP-1), and IL-27. CHI3L1 is an inducer of the PI3K/AKT signaling pathways [29] and M2 mature differentiation of macrophages for Th2 inflammation [30], IL-6 and MCP-1 are inducers of M2 mature differentiation of macrophages [31], and IL-27 is a cytokine involved in anti-inflammatory and immune-regulatory functions and inhibits Th2, innate lymphoid cell-2 (ILC2), and Th17 responses [32]. Moreover, compared to 2D hUCB-MSC-derived EVs, there was decreased expression of dickkopf 1 (Dkk-1), an inhibitor of Wnt signaling [33–35]. Wnt/*β*-catenin plays a role in the development and function of insulin-producing *β*-cells [36]. These differences in protein expression profiles might have contributed to the greater protective effects of 3D hUCB-MSC-derived EVs on islet inflammation and dedifferentiation in this study.

Although the results of our study are in line with previous research suggesting the superior immunomodulatory effects of 3D MSCs over 2D MSCs [15, 16, 18, 22], some previous studies reported contradictory results [19, 37]. In one study, T cell-suppressive abilities of MSCs were observed only in 2D MSCs and not in 3D MSCs; in that study, the T cell-suppressive abilities of 3D MSCs were partly restored by addition of a corticosteroid [37]. Another previous study compared the immunomodulatory potency of 2D MSC-derived EVs and 3D MSC-derived EVs *in vitro* and then compared their anti-inflammatory and antifibrotic potentials *in vivo* using a bleomycin-induced lung fibrosis model [19]. In that study, the *in vitro* immunomodulatory potency of 2D MSC-derived EVs and 3D MSC-derived EVs was compared after IFN-*γ* stimulation. The T cell suppression ability in terms of indoleamine 2,3-dioxygenase activity after IFN-*γ* stimulation and macrophage phenotype in terms of phagocytosis activity were lower in 3D MSC experiments, indicating polarization into the M1 subtype, although they did not directly measure M1 or M2 markers on macrophages [19]. In our study, the M2-polarizing ability of 3D MSCs was optimized when 3D MSCs were generated with sufficient cell numbers, without hypoxia or cytokine stimulation. In 25 K 3D MSCs, cytokine stimulation reduced the M2-polarizing ability. Therefore, it is possible that the immunomodulatory properties of 3D MSC-derived EVs could be lost if 3D culture conditions are not optimized. We also used a different source of MSCs (human UCB-MSCs in the current study vs. human lung tissue-derived MSCs or bone marrow-derived MSCs in the previous study [19]) and different disease models (serum-deprived culture of isolated pancreatic islets and cocultivation of hIAPP-producing pancreatic islets, MSCs, and macrophages). In this context, it is reassuring that the uniform-sized 3D MSCs produced by nanopatterned culture plasticware prevented *β*-cell death in a multiple low-dose streptozotocin-induced diabetes model [22], in which immune cell infiltration and progressive loss of *β*-cells typically occurs. Whether 3D MSC-derived EVs alone without the cellular component would have similar benefit should be explored in future research.

An important translational potential in our study is the use of EVs derived from 3D hUCB-MSCs in the pretransplant cultures of isolated human islets before clinical islet transplantation. Our previous study showed that serum deprivation, as in pretransplant cultures in clinical intraportal islet transplantation to avoid the use of animal-derived materials, induces an inflammatory response in cultured hIAPP^+/-^ islets even without the prolonged culture with hyperglycemia [24]. This study hypothesized that the addition of EVs derived from 3D hUCB-MSCs during the pretransplant culture of islets could attenuate the inflammatory response and loss of *β*-cell identity caused by serum deprivation. In this study, 3D hUCB-MSC-derived EVs promoted the M2 polarization of islet-resident macrophages and reduced the inflammasome activation induced by serum deprivation in hIAPP heterozygote mouse islets. This resulted in improved islet viability and insulin response to glucose, attenuation of proinflammatory cytokine expression, and preservation of *β*-cell identity. In addition to the enhanced M2 polarization ability of 3D hUCB-MSC-derived EVs, this therapeutic effect could result from differences in the cargo and the proteome profiles of EVs derived from 2D and 3D hUCB-MSCs, which include several angiogenesis-related cytokines.

Several limitations of this study should be discussed. First, the 3D culture condition has not been optimized in experiments with primates or human islets using 3D hUCB-MSC-derived EVs. The concentration of 3D hUCB-MSC-derived EVs should be optimized using human islets before clinical application. Second, whether the results of this study can be reproduced by the systemic use of 3D hUCB-MSC-derived EVs in type 2 diabetes or islet transplant recipients remains unclear. Third, we assessed only a limited number of miRs that promoted M2 polarization of macrophages in a previous study using cytokine-pretreated 2D MSCs, rather than a systemic comparison of all miRs related to macrophage M1/M2 polarization. Fourth, we did not investigate whether 3D MSC-derived EVs could modulate immune responses of T cells and other components of immune system as well. Although we speculate that 3D MSC-derived EVs might be helpful for prevention of islet graft rejection by alloimmune responses as well, the data presented in this study is insufficient to support the hypothesis.

In conclusion, we found that EVs derived from 3D hUCB-MSCs can protect hIAPP-expressing pancreatic islets against nonspecific inflammation and dedifferentiation, and these benefits were significantly greater than those of 2D hUCB-MSC-derived EVs. These results might be, at least in part, due to the enhanced M2 polarization ability of the EVs derived from 3D hUCB-MSCs compared to those derived from 2D hUCB-MSCs on islet-resident macrophages.

## Data Availability

The datasets during the current study are available from the corresponding authors on reasonable request.

## Supplementary Materials

Supplementary Materials Table S1: sequences of gene-specific primer pairs used for real-time qRT-PCR. Figure S1: uncropped western blot images used in this study. Figure S2: flow cytometry analysis of M2 polarization of pancreatic macrophages by 3D hUCB-MSC-derived extracellular vesicles (EVs). Figure S3: 3D hUCB-MSC-derived extracellular vesicles (EVs) and M2 polarization of THP-1 monocytes.
