# Supplementary material for: Extracellular Vesicles Derived from Three-Dimensional-Cultured Human Umbilical Cord Blood Mesenchymal Stem Cells Prevent Inflammation and Dedifferentiation in Pancreatic Islets
Source: Stem Cells Int. 2023 Feb 20;2023:5475212. doi: 10.1155/2023/5475212 (PMC9970714; doi:10.1155/2023/5475212)
Supplement: Supplementary Materials — Table S1: sequences of gene-specific primer pairs used for real-time qRT-PCR. Figure S1: uncropped western blot images used in this study. Figure S2: flow cytometry analysis of M2 polarization of pancreatic macrophages by 3D hUCB-MSC-derived extracellular vesicles (EVs). Figure S3: 3D hUCB-MSC-derived extracellular vesicles (EVs) and M2 polarization of THP-1 monocytes. [file 5475212.f1.zip › Supplementary Materials_16Dec2022.docx]

## Supplementary Materials

Table S1: Sequences of gene-specific primer pairs used for real time qRT-PCR

| Gene | | Accession number | Primer Sequence (5'-3') | Reference |
| --- | --- | --- | --- | --- |
| TNF-α | | BC137720.1 | Forward: 5′- CTGAACTTCGGGGTGATCGG -3′  Reverse: 5′- GGCTTGTCACTCGAATTTTGA -3′ | [1] |
| IL-1β | | BC011437.1 | Forward: 5′- TGGAAAAGCGGTTTGTCT -3′  Reverse: 5′- ATAAATAGGTAAGTGGTTGCC -3′ |  |
| IL-18 | | NM_008360.2 | Forward: 5′- TGGTTCCATGCTTTCTGGACTCCT -3′  Reverse: 5′- TTCCTGGGCCAAGAGGAAGTGATT -3′ |  |
| NLRP3 | | NM_145827.4 | Forward: 5′- TGCTCTTCACTGCTATCAAGCCCT -3′  Reverse: 5′- ACAAGCCTTTGCTCCAGACCCTAT -3′ | [2] |
| HMGB1 | | BC110667.1 | Forward: 5′- CGAGAGGCAAAATGTCCTCA -3′  Reverse: 5′- TCATAACGAGCCTTGTCAGC -3′ |  |
| caspase-1 | | NM_009807.2 | Forward: 5′- AGATGCCCACTGCTGATAGG -3′  Reverse: 5′- TTGGCACGATTCTCAGCATA -3′ | [3] |
| Oct4 | | NM_013633.3 | Forward: 5′- AAACCGTCCCTAGGTGAGCC -3′  Reverse: 5′- GAGTTGCTTTCCACTCGTGC -3′ |  |
| NGN3 | NM_009719.6 | | Forward: 5′- GGTAGCACTACCTAGTTGGAGACTC -3′  Reverse: 5′- GACAAACAGTGCTTCAGGAACCGTC -3′ |  |
| FoxO1 | | NM_019739.3 | Forward: 5′- CGGAAAATCACCCCGGAGAA -3′  Reverse: 5′- TACACCAGGGAATGCACGTC -3′ |  |
| Pdx1 | | NM_008814.4 | Forward: 5′- CCACCCCAGTTTACAAGCTC -3′  Reverse: 5′- TGTAGGCAGTACGGGTCCTC -3′ |  |
| β-actin | | NM_007393.5 | Forward: 5′- TGTTACCAACTGGGACGACA -3′  Reverse: 5′- GGGGTGTTGAAGGTCTCAAA -3′ | [1] |

TNF-α, Tumor necrosis factors-alpha; IL-1β, Interleukin-1 beta; IL-18, Interleukin 18; NLRP3, NOD-like receptor pyrin domain-containing protein 3; HMGB1, High Mobility Group Box 1; NGN3, Neurogenin 3; FoxO1, Forkhead box protein O1; Pdx1, pancreatic and duodenal homeobox 1

**
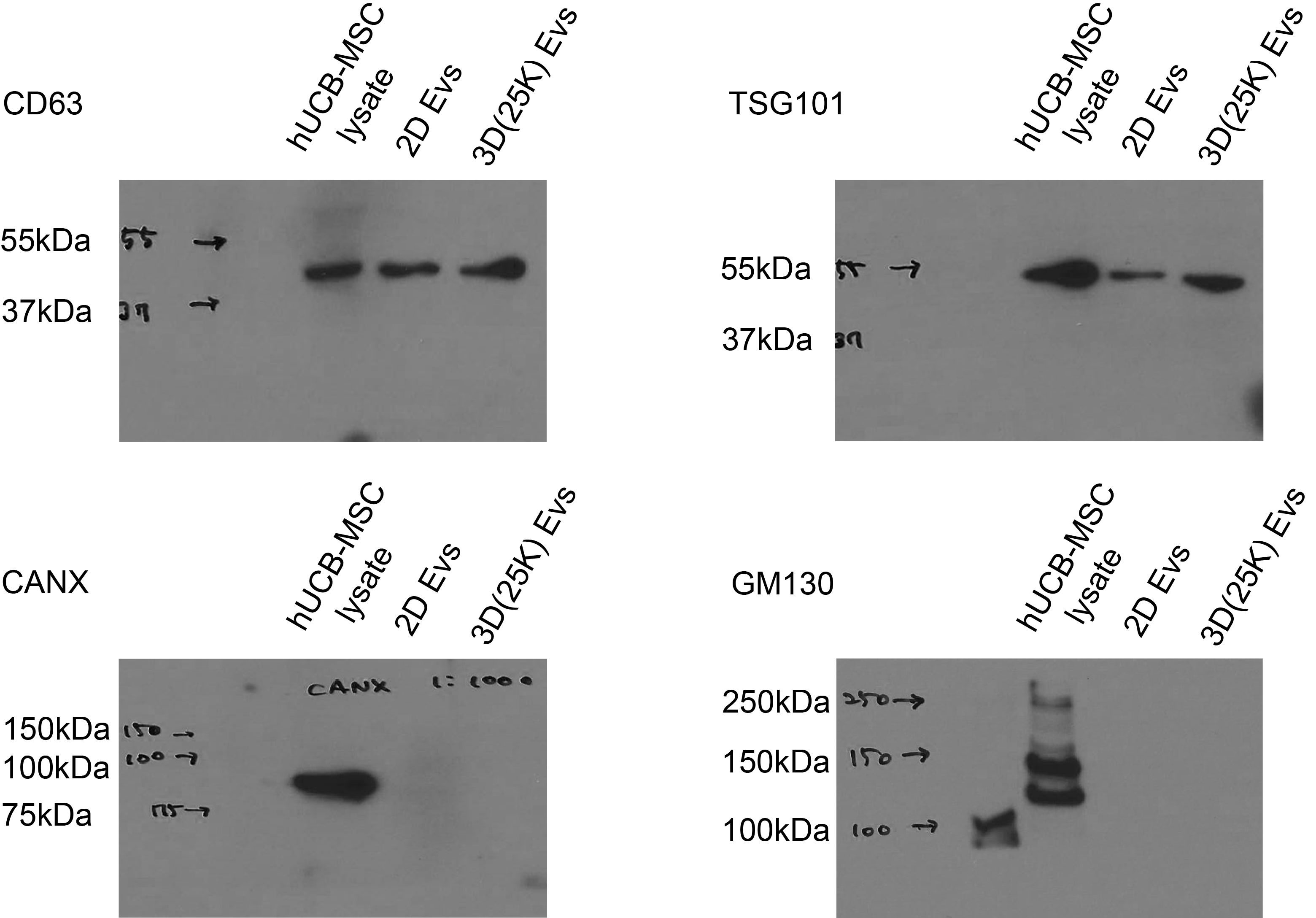
**

Figure S1: Uncropped western blot images used in this study. Molecular weights are presented in kDa units.

##
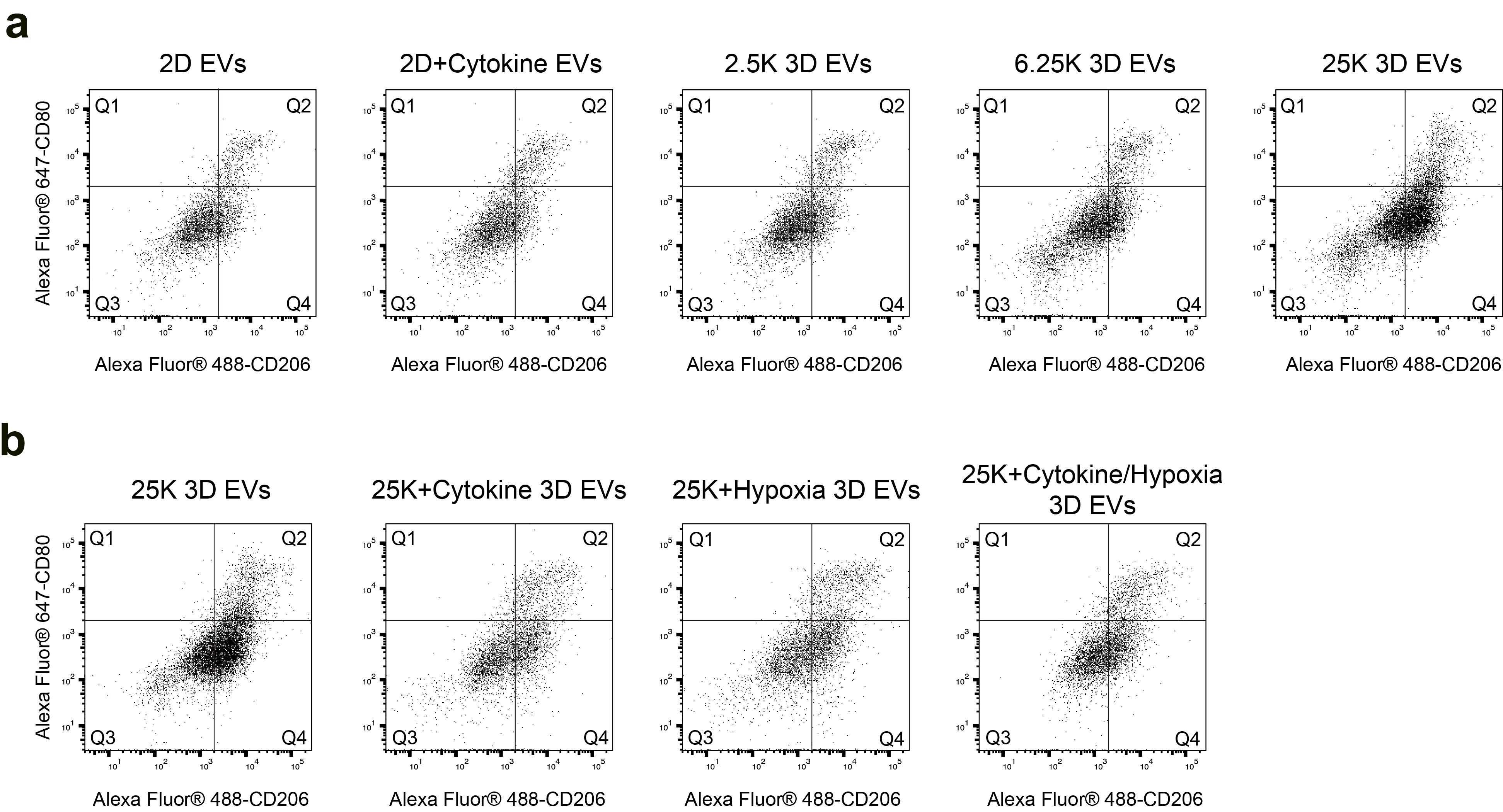


Figure S2: Flow cytometry analysis of M2 polarization of pancreatic macrophages by 3D hUCB-MSC-derived extracellular vesicles (EVs). (a) Original images for dot plot of flow cytometry analysis of cell surface molecules CD80 and CD206 on pancreatic macrophages cultured with monolayer (2D) and 3D hUCB-MSC-derived EVs. (b) Original images for dot plot of flow cytometry analysis of cell surface molecules CD80 and CD206 on pancreatic macrophages cultured in the presence of EVs isolated from the supernatants of 25K 3D hUCB-MSCs unstimulated or preconditioned with hypoxia or cytokines (TNF-α and IFN-γ, each 40 ng/mL).


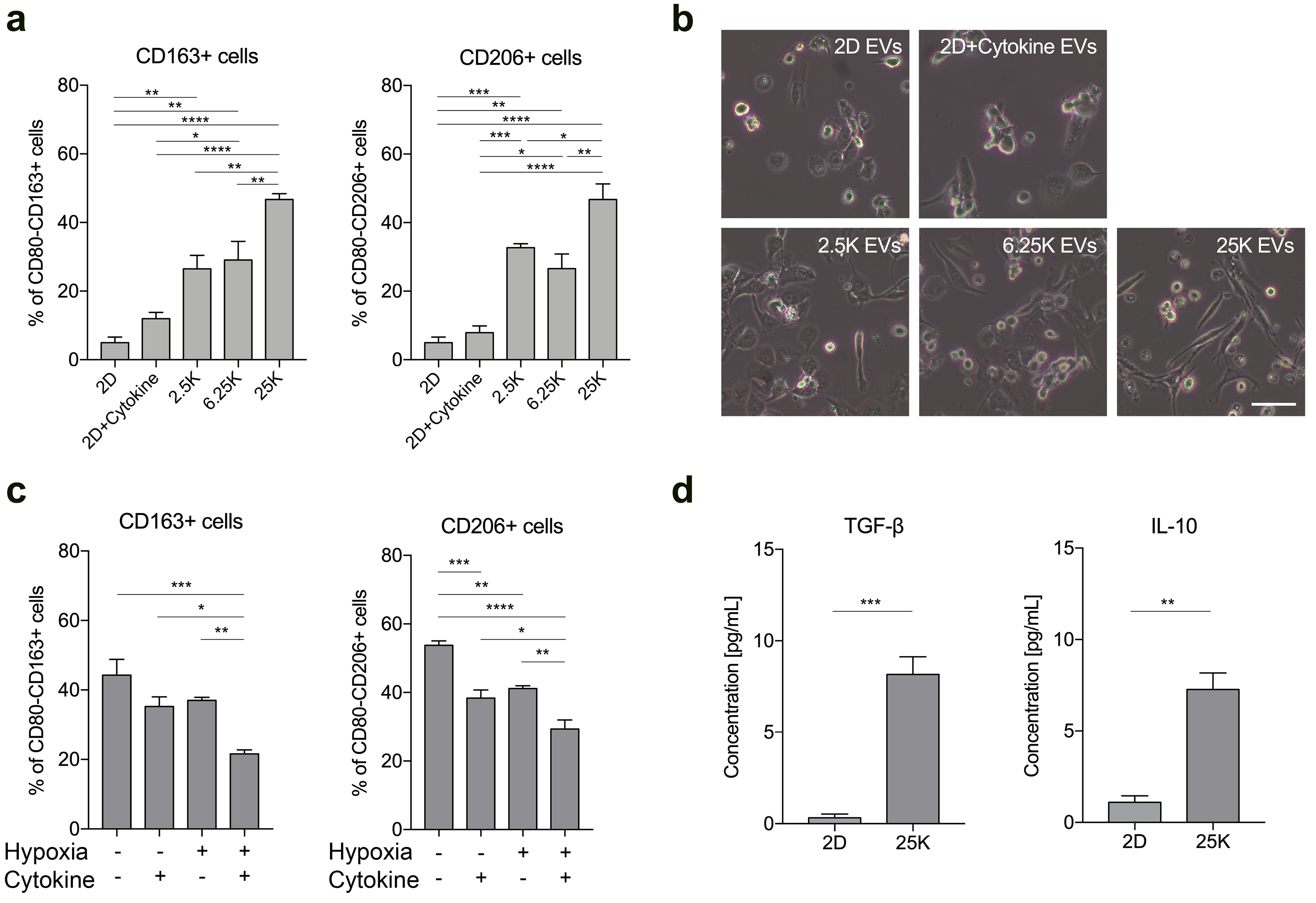


Figure S3: 3D hUCB-MSC-derived extracellular vesicles (EVs) and M2 polarization of THP-1 monocytes. (a) Flow cytometry analysis of cell surface molecules CD163, CD206, and CD80 on THP-1 monocytes cultured with monolayer (2D) and 3D hUCB-MSC-derived EVs for 48h. (b) Representative phase-contrast microscopic images (100× magnification) of THP-1 monocytes cultured in the presence of EVs isolated from the supernatants of 2D or 3D-cultured (2.5K, 6.25K, and 25K) hUCB-MSCs. Scale bar=200 μm. (c) Flow cytometric analysis of cell surface molecules CD163, CD206, and CD80 on THP-1 monocytes cultured in the presence of EVs isolated from the supernatants of hUCB-MSCs that were unstimulated or preconditioned with hypoxia or cytokines (TNF-α and IFN-γ, each 40 ng/mL). For preconditioning with cytokines, 3D-cultured hUCB-MSCs were seeded into a Petri dish, after 24 h culture medium was replaced with fresh culture medium supplemented with 10% exosome-depleted FBS (Gibco) with recombinant human interferon-gamma (IFN-γ; Peprotech, Rocky Hill, NJ, USA) and tumor necrosis factor-alpha (TNF-α; Peprotech) at a final concentration of 40 ng/ml. (d) Medium concentrations of TGF-β and IL-10 were measured by ELISA. Columns, mean; bars, SD, **p <* 0.05, ***p <* 0.005, ****p <* 0.0005 and *****p <* 0.0001, by one-way ANOVA and Tukey’s post-test.

## References

1. Kim G, Lee HS, Oh BJ, Kwon Y, Kim H, Ha S, et al. Protective effect of a novel clinical-grade small molecule necrosis inhibitor against oxidative stress and inflammation during islet transplantation. Am J Transplant. 2021;21(4):1440-52.

2. Kaushik DK, Gupta M, Kumawat KL, Basu A. NLRP3 inflammasome: key mediator of neuroinflammation in murine Japanese encephalitis. PLoS One. 2012;7(2):e32270.

3. Sun S, Xia S, Ji Y, Kersten S, Qi L. The ATP-P2X7 Signaling Axis Is Dispensable for Obesity-Associated Inflammasome Activation in Adipose Tissue. Diabetes. 2012;61(6):1471-8.
