# Supplementary figures and images for: Extracellular Vesicles Derived from Three-Dimensional-Cultured Human Umbilical Cord Blood Mesenchymal Stem Cells Prevent Inflammation and Dedifferentiation in Pancreatic Islets
Source: Stem Cells Int. 2023 Feb 20;2023:5475212. doi: 10.1155/2023/5475212 (PMC9970714; doi:10.1155/2023/5475212)

**a**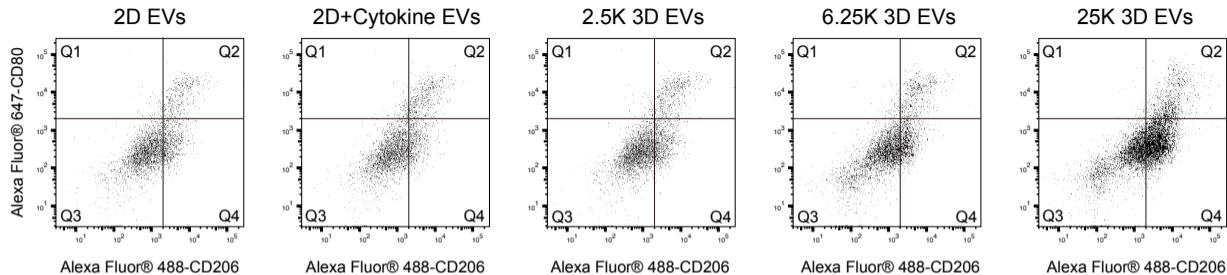**b**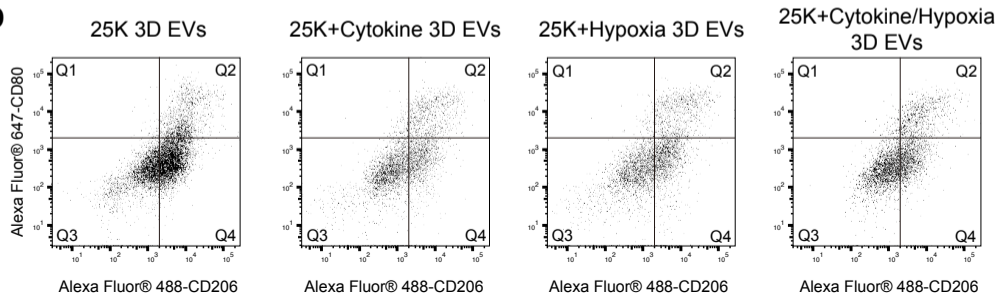

Supplement: Supplementary Materials — Table S1: sequences of gene-specific primer pairs used for real-time qRT-PCR. Figure S1: uncropped western blot images used in this study. Figure S2: flow cytometry analysis of M2 polarization of pancreatic macrophages by 3D hUCB-MSC-derived extracellular vesicles (EVs). Figure S3: 3D hUCB-MSC-derived extracellular vesicles (EVs) and M2 polarization of THP-1 monocytes. [file 5475212.f1.zip › Figure S2.pdf]

**a**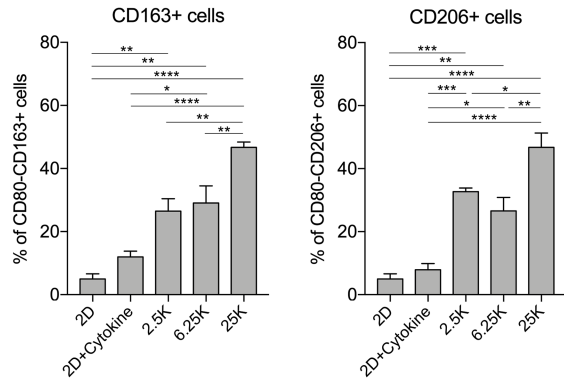**b**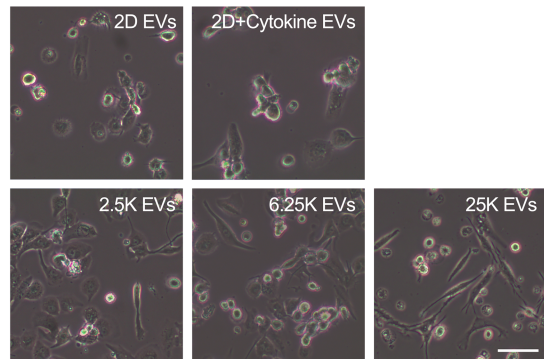**c**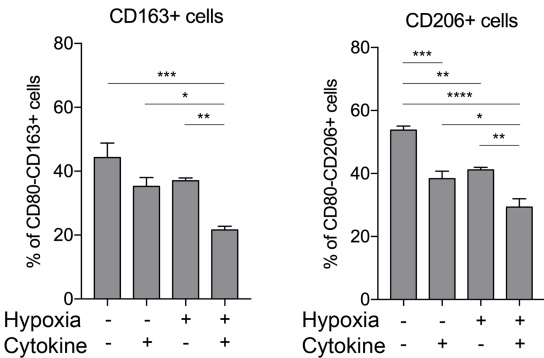**d**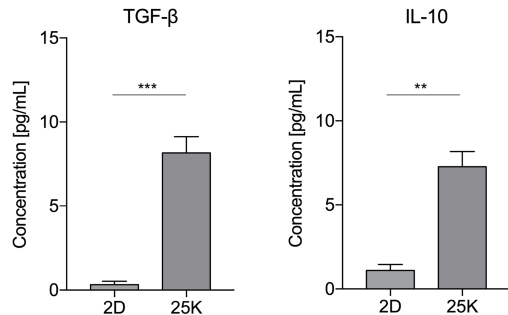

Supplement: Supplementary Materials — Table S1: sequences of gene-specific primer pairs used for real-time qRT-PCR. Figure S1: uncropped western blot images used in this study. Figure S2: flow cytometry analysis of M2 polarization of pancreatic macrophages by 3D hUCB-MSC-derived extracellular vesicles (EVs). Figure S3: 3D hUCB-MSC-derived extracellular vesicles (EVs) and M2 polarization of THP-1 monocytes. [file 5475212.f1.zip › Figure S3.pdf]
